# Retinal Development and Ommin Pigment in the Cranchiid Squid *Teuthowenia pellucida* (Cephalopoda: Oegopsida)

**DOI:** 10.1371/journal.pone.0123453

**Published:** 2015-05-13

**Authors:** Aaron B. Evans, Monica L. Acosta, Kathrin S. Bolstad

**Affiliations:** 1 Institute for Applied Ecology New Zealand, Auckland University of Technology, Auckland, New Zealand; 2 Department of Optometry and Vision Science, Auckland, New Zealand; 3 New Zealand National Eye Centre, The University of Auckland, Auckland, New Zealand; University Zürich, SWITZERLAND

## Abstract

The cranchiid *Teuthowenia pellucida*, like many deep-sea squid species, possesses large eyes that maximise light sensitivity in a nearly aphotic environment. To assess ontogenetic changes in the visual system, we conducted morphometric and histological analyses of the eyes using specimens from New Zealand collections. While the ratio between eye diameter and mantle length maintained a linear relationship throughout development, histological sections of the retina revealed that the outer photoreceptor layer became proportionally longer as the animal aged, coincident with a habitat shift into deeper, darker ocean strata. Other retinal layers maintained the same absolute thickness as was observed in paralarvae. Granules of the pigment ommin, normally located in the screening layer positioned at the base of the photoreceptors, were also observed at the outer end of the photoreceptor segments throughout the retina in young and mid-sized specimens. Early developmental stages of this species, dwelling in shallow waters, may therefore rely on migratory ommin to help shield photoreceptors from excess light and prevent over-stimulation. The oldest, deeper-dwelling specimens of *T*. *pellucida* examined had longer photoreceptors, and little or no migrated ommin was observed; we suggest therefore that short-term adaptive mechanisms for bright light conditions may be used primarily during epipelagic, early life stages in this species.

## Introduction

Deep-sea squids successfully live and feed in dark oceanic habitats, with vision apparently playing an important role in gathering environmental information [1]. Squid possess a camera-type eye and are divided into two suborders according to the presence of an external “cornea”: in myopsids, a membrane develops from tissue behind the eyeball and grows outward to cover the eye and lens [2], while oegopsid squid eyes lack this covering and the lens is in direct contact with the surrounding water. In the eyes of both suborders, a lens (formed by two hemispherical halves), suspended in place by ciliary muscles, focuses the light onto a simple non-inverted retina [3]. Projected light is inverted by the circular lens; therefore, the inferior retina processes down-welling light, while the superior retina processes upwelling light [3]. In basic structure and light sensitivity [3], cephalopod eyes thus bear astonishing resemblance to vertebrate eyes, given that they share no common ancestor capable of complex vision and visual processing. These eyes are a striking example of convergent evolution, especially given that the eyes of cephalopods develop as invaginations of the skin midway through embryonic growth, while vertebrate eyes develop as extrusions of the central nervous system [4]. While some investigation has been conducted into cephalopod vision [4–6], existing literature has largely focused on visual capabilities in octopuses, and many anatomical details of squids’ ocular structures, apart from those of the coastal myopsid *Doryteuthis pealeii*, remain largely unknown.

Within the squid retina, there are photoreceptor cells and supporting cells [6]. The photoreceptor contains visual pigments in the outer region that react to incoming light, and the signal is transmitted to the brain through changes in the membrane potential in the inner region. Photoreceptor length (combined inner and outer regions) varies within the squid retina: those found towards the ventral part of the retina (processing down-welling light in the ‘normal’ swimming orientation for most squids) tend to be longer than those found on the dorsal region [7]. The photoreceptor cells contain a darkly pigmented screening layer between the inner and outer segments, preventing the majority of light from entering the inner segment. In cephalopods, this screening pigment has been identified as ommin (Butenandt, as cited in [5]), and has been shown to migrate between its “natural” position in the screening layer at the base of the outer photoreceptor segment, and the outward tip of the outer segment, depending on light conditions [5, 6]. This physiological process prevents over-stimulation of the photoreceptors; similar screening pigments have been studied in other invertebrates [8], but only in a handful of cephalopods, and these mostly octopuses [6, 9], although Daw and Pearlman conducted initial investigations into squid ommin migration using the myopsid *Doryteuthis pealeii* [5].

While examining ontogenetic series of the cranchiid (or “glass”) squid *Teuthowenia pellucida* from New Zealand collections [10], a number of specimens were encountered with well-preserved eyes. This material provided a rare opportunity to examine eye anatomy in a deep-sea squid, since the often large, delicate eyes of cephalopods are commonly damaged or destroyed during the collection process. *Teuthowenia pellucida* is a particularly interesting visual subject in that it spends its early life (paralarval and juvenile) stages in shallow, brightly lit waters (above 200 m), and later descends to dwell at great depth, some as deep as two kilometers [10, 11], where bioluminescence forms the only light source [1]. This species’ evident retention of effective vision across such disparate light conditions is intriguing. Therefore, observations on the eye’s structure and growth were made, to seek insight into the developmental changes that appear to enable this squid to maintain effective vision across a lifespan carried out in first photic, then aphotic waters.

## Materials and Methods

A total of 87 specimens of *T*. *pellucida* captured at known depths (ranging in size from 1.5 to 210 mm dorsal mantle length—ML) were loaned from the National Institute of Weather and Atmospheric Research, Ltd (NIWA) and the National Museum of New Zealand Te Papa Tongarewa (NMNZ) in Wellington, New Zealand. Thirty of the specimens were adults, with 23 reproductively mature or mated. Although geographic and depth data were available, light conditions during capture (i.e., day/night) were not. Of these specimens, 61 possessed intact enough eyes to permit measurement, and seven in particularly good condition were selected for histological examination.

### Ethics

Ethics approval was not required for this study as no live animals were used for this research.

### Tissue Preparation

Whole eyes were removed from seven preserved specimens of *Teuthowenia pellucida*, which had been fixed in 5% buffered formalin and stored in 70–80% ethanol until processed. The eyes were washed several times in 0.1M Phosphate Buffer Saline (PBS) solution (pH 7.4) for 24 hours, before being submerged in 30% sucrose dissolved in PBS overnight. Eyes were either hemisected along the ventro—dorsal midline, vertically sectioning the lens in two, or were sectioned along the equator to obtain dorsal and ventral sections. Tissues were then embedded in TissueTek cryo-OCT compound (Fisher Scientifics) and frozen at -20°C before sectioning retinal samples using a LEICA cryostat (LEICA, Germany) set at a thickness of 16–20 μm. Eye sections were transferred to glycerine-coated slides and were stored at -20°C until stained.

Tissue sections were stained using a standard haematoxylin and eosin (HE) staining procedure for contrast of nuclei and cytoplasm, or were stained in fuchsin. The slides were washed in PBS, hydrated and placed into absolute ethanol for five minutes. The slides were then placed in a staining solution prepared by combining distilled water (730 ml), glacial acetic acid (20 ml), aluminum sulphate (17.6 gr), sodium iodate (0.2 gr), ethylene glycol (250 ml) and haematoxylin (2 gr) for five minutes. Slides were then washed under running distilled water and stain differentiated in 1% acid alcohol. After rinsing, the slides were dipped in 1% lithium carbonate and then washed in distilled water for five minutes. 1% eosin stain was used for contrast. Following staining, the slides were rinsed under distilled water and then rinsed consecutively in 95% ethanol, xylene, and mounted with DPX mounting medium (Sigma).

Stained samples were visualised using a LEICA DR2 bright field microscope. Images were captured using a LEICA DC 500 digital camera. A standard stage micrometer was used to calibrate the optical images.

The eye diameter and mantle length were measured using digital callipers for specimens smaller than 10 mm ML and a standard ruler for larger specimens. Values were entered into a Microsoft Excel spreadsheet and a scatter-plot graph was constructed. The Data Analysis tool was used in the construction of the best fit regression line.

Retinal sections were analysed and measured using the calibrated ruler superimposed onto the images at each magnification. The measurements were taken in central and peripheral areas of the retina (each location measured three times and averaged), from the junction of the outer segment with the posterior chamber, to the end of the support cell layer.

## Results

### Relative eye size throughout ontogeny

**Fig 1:**
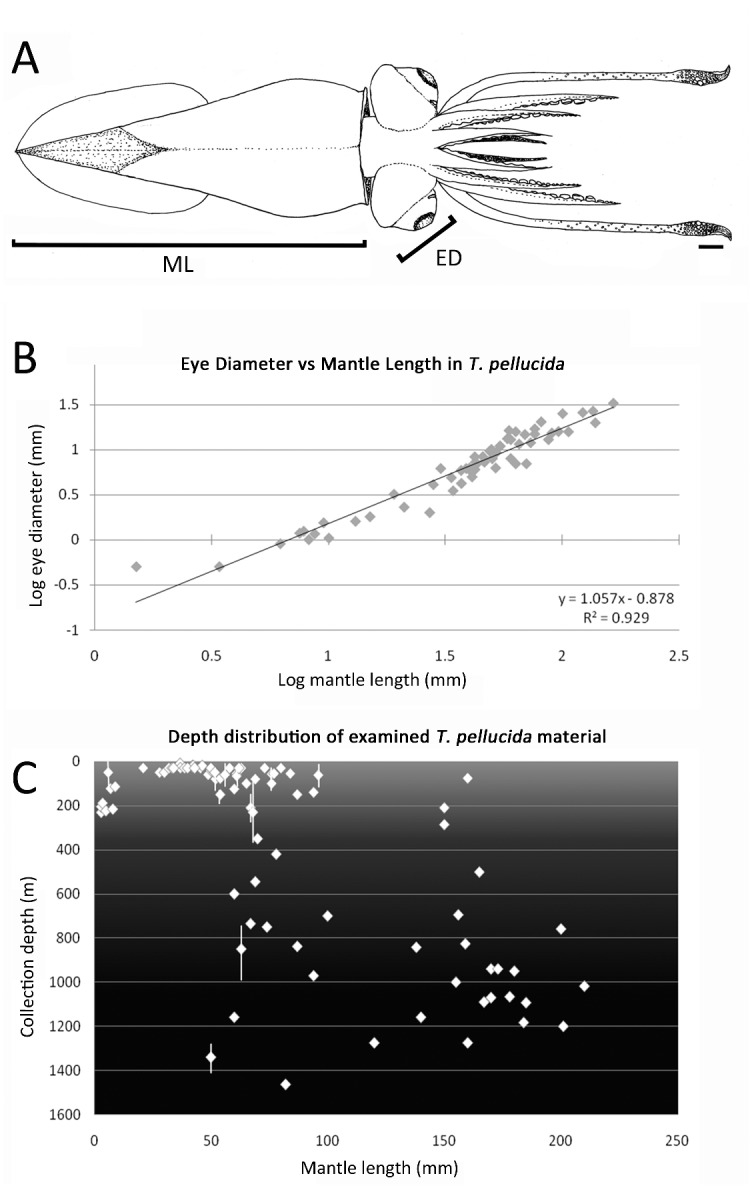
(A) Schematic of *T*. *pellucida*, indicating measurements for mantle length (ML) and eye diameter (ED). (B) Log relationship between ML and ED. (C) Depth distribution of the 87 specimens examined in this study with known collection strata, plotted against ML.

Ontogenetic changes in eye-to-body-size ratio were investigated by measuring the eye diameter along the dorsal—ventral midline (0.5–33 mm) and mantle length (ML 1.5–165 mm) for specimens with intact eyes (Fig 1A). These two measurements maintained a linear growth ratio throughout ontogeny; Fig 1B shows the relationship plotted as the natural log of the raw values (R^2^ value = 0.929). In specimens below ML 50 mm (n = 33), a close correlation between eye diameter and mantle length was observed, while greater variation was observed in larger specimens (n = 28). Beginning around ML 50 mm, a positive correlation was observed between body size and depth of capture (Fig 1C).

### Retinal layers

**Fig 2:**
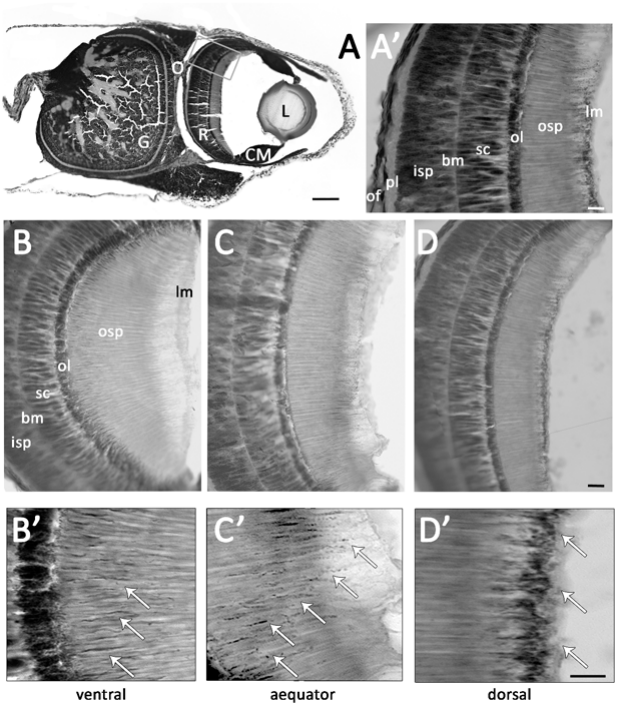
(A) Cross-section of larval (ML 9.7 mm) *T*. *pellucida* eye, with insert (A’) showing retinal layers. (B–D) Changes in retinal thickness observed in serial ventral, equatorial and dorsal sections through retina of same specimen, with location of main ommin layer and migrated ommin granules shown in high magnification in B’–D’ (migrated granules indicated by white arrows). Abbreviations: basal membrane (bm), ciliary muscle (CM), ganglia (G), inner segment of photoreceptors (isp), lens (L), limiting membrane (lm), ommin layer (ol), optic nerve (O), optic fibers (of), outer segment of photoreceptors (osp), plexiform layer (pl), retina (R), supporting cells (sc). Scale bars = (A) 100 μm, all other images 10 μm.

The structure of the retina was examined for seven specimens (ML 9.7–102.0 mm, Table 1) representing progressive ontogenetic stages (stages A, C, E, and F, as outlined by Evans [10]). In all stages, the retina is connected to an optic ganglion through the wide optic nerve. In young specimens, retinal cross sections showed a thin anterior limiting membrane in contact with the striated outer segment of the photoreceptor (striation visible only at high magnifications); a thin, dark segment (ommin layer, the screening pigment); and the nuclei of supporting cells resting on a basal membrane that partitioned them from the inner segment of the photoreceptors. A plexiform layer was also observed between the inner segment of the photoreceptor and the axons, which form the most internal layer of the retina (Fig 2). Ontogenetic differences were noted in the thicknesses of several retinal layers (see Table 1): in younger specimens (*e*.*g*., M.070961) the outer and inner segments of each region were of nearly equal length (often differing by just 10 μm), while in older specimens, the outer segment was significantly longer than the inner and supporting cells(N = 9, p<0.0001). In general, photoreceptors in the ventral region of the retina also appeared longer than those found in the dorsal region (Fig 2B and 2D), although this was not tested statistically.

**Table 1:** Measurements of visible retinal layers in the ventral retina of *Teuthowenia pellucida*.

Number	ML	Location	OS	Ommin Band	IS	Supporting Cells	PR	Total Retina	OS/IS	PR/ Retina
NMNZ M.070961	9.7 mm	P	30	10	35	30	75	105	0.85	0.71
NMNZ M.070961	9.7 mm	C	50	10	40	50	100	150	1.25	0.66
NMNZ M.287201	27.0 mm	P	110	5	70	65	185	250	1.57	0.74
NMNZ M.287201	27.0 mm	C	75	5	70	70	150	220	1.07	0.68
NIWA 71673	50.0 mm	P	130	10	15	45	155	200	8.66	0.77
NIWA 71673	50.0 mm	C	190	10	20	60	220	280	9.50	0.78
NMNZ M.074310	52.0 mm	P	220	10	10	60	240	300	22.00	0.80
NMNZ M.074310	52.0 mm	C	220	10	15	80	245	325	14.66	0.75
NMNZ M.067262	52.0 mm	P	185	10*	20	20	205	225	9.25	0.91
NMNZ M.067262	52.0 mm	C	290	10*	35	100	325	425	8.28	0.76
NIWA 71673	102.0 mm	C	360	10	10	60	380	440	36.00	0.86
NIWA 71691	128.0 mm	P	290	10	25	50	325	375	11.60	0.86
NIWA 71691	128.0 mm	C	340	10	30	50	380	430	11.33	0.88

“Location” indicates region in which the photoreceptors were measured: central portion of the equatorial retinal (C) or in the periphery (P). The photoreceptor outer segment (OS), ommin band, and inner photoreceptor segment (IS) make up the total photoreceptor length (PR), which ranged from 75–380 μm. Total retinal thickness refers to the length of the supporting cell layer and photoreceptors together (105–440 μm).

* Asterisk indicates an approximation due to damaged layers. Retinal measurements in micrometres (μm).

**Fig 3:**
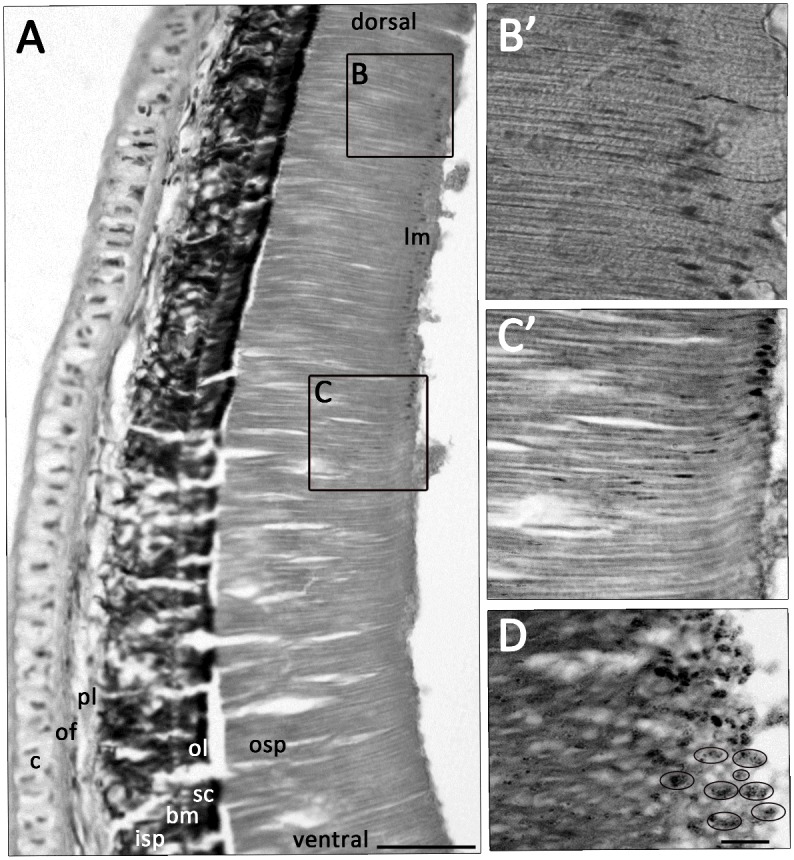
(A) Cross-section of adult (ML 52 mm) *T*. *pellucida* retina showing retinal layers and progressive dorsal—ventral lengthening of outer photoreceptor segment. Migrated ommin pigment in dorsal (B) and equatorial (C) regions and representative areas are shown in high magnification (B’, C’). (D) Oblique section through outer photoreceptor segment shows that migrated ommin pigment is located between photoreceptor cells; black circles indicate areas of clustered pigment granules. Abbreviations: rudimentary cartilage (c). All other abbreviations as in Fig 2. Scale bars = (A) 100 μm, (B’–D) = 10 μm.

In paralarval and juvenile specimens (ML ≤ 50mm, n = 2), the ommin granules were distributed in two layers in the dorsal portion of the retina: a dense, dark layer between the inner and outer segments of the photoreceptors, and a sparser secondary layer at the outer end of the photoreceptor (Fig 2D’). Ventrally, ommin pigment granules were concentrated close to the main screening layer (Fig 2B’) but in the central equatorial region, some granules were observed in transit between the main ommin layer and the tip of the photoreceptors (Fig 2C’), in all three of the young specimens examined. In specimens above ML 50 mm (n = 5), the outer segment of all photoreceptors was longer than the inner segment (Fig 3A), and photoreceptors were noticeably longer in the ventral region of the retina than in the dorsal region. In mid-size specimens (ML 50–52 mm), the ommin granules appeared to have migrated toward the outer surface of the retina at the equator of the eye, and were sparsely scattered throughout the outer portion of the ventral part of the retina (Fig 3C’), while in the largest eyes analysed (from specimens of ML 102, 128 mm), all pigment granules were positioned within the main ommin layer. An oblique section through the outer segment of the photoreceptors in an eye with migrated ommin (NIWA 71673; Fig 3D) showed that the pigment granules appeared to be interspersed with, but exterior to, the photoreceptor cells themselves.

## Discussion

This study reports three interesting phenomena: (1) Throughout the animal’s development, the eye diameter of *T*. *pellucida* maintains a linear growth rate relative to the body size (ML); (2) an ontogenetic descent into aphotic waters seems to be associated with significant proportional enlargement of the outer segment of the photoreceptors, especially in the ventral portion of the retina (which receives down-welling light, with (3) further adaptation to light enabled by migration of the ommin granules’ position.

### Eye-size-to-body ratio

Many animals that rely on vision in the mesopelagic deep sea have proportionally large eyes [1]; the cephalopod orders Teuthida and Vampyromorpha are excellent examples. The vampire squid, *Vampyroteuthis infernalis*, has the largest eye-diameter-to-total-body-length ratio of any animal (about 1:6, 1:3–1:4 to mantle length) [12]. The largest known eyes in the animal kingdom belong to the colossal squid (*Mesonychoteuthis hamiltoni*, also in the family Cranchiidae), and attain at least 27–35 cm in diameter ([13]; pers. obs.). However, these eyes, while large in absolute terms, are about half the relative size (eye diameter about 10% ML, and 2–4% total body length, pers. obs.) of those in *T*. *pellucida*. Large, visually oriented predatory fish such as the swordfish (*Xiphias gladius*) have a similar eye-diameter-to-body-length ratio (about 2–3% at mature sizes of 3–5m; [13]) although their skulls could support much larger eyes, suggesting that, beyond a certain threshold, these large vertebrates are similarly subject to the law of diminishing returns in visual acuity with increased eye size [13]. *Teuthowenia pellucida*, however, which matures around 100 mm ML [10] likely falls within a body (and eye) size range throughout its lifespan where the extra energy expended in maximising eye size provides a worthwhile return in increased visual sensitivity; certainly the proportion of the head comprised of ocular tissue (about 88% by volume in sub-adults, pers. obs.) appears extreme.

Among cephalopods, the visual capabilities of those living in the deep sea are the least understood, in part because many of their tissues (including eye and nervous tissues) are often gelatinous and fragile, and severely damaged or destroyed entirely during capture [14]. *Teuthowenia* is no exception; the large, delicate eyes (observed diameter up to 33 mm, making the head width sometimes greater than the mantle width) were badly damaged on most of the material examined herein, permitting detailed histological examination of only a few specimens. When intact, these eyes appeared disproportionately large relative to the body, especially in sub-mature to mature individuals; however, observations made throughout ontogeny show a linear ratio of eye diameter to mantle length—about 1:5 (Fig 1B). Additional material, especially at the larger end of this species’ size range (reported to attain ML of 200+ mm, while the largest specimen examined herein was ML 165 mm) would be useful in examining this trend.

Voss reported that *Teuthowenia*, like other cranchiid squids, undergoes an ontogenetic vertical migration to the deep sea [11], an observation supported by collection data for the material examined herein (Fig 1C), and many cranchiid species are thought to migrate diurnally in the water column [15]. At the shallower depths occupied by paralarvae and juveniles, the eyes would be subjected to much higher light intensities, while larger eyes (in absolute terms) would maximise light sensitivity as the animal matures and descends into the aphotic zone. Perhaps the more surprising finding from the eye-size component of this study is therefore not that the adults have a relatively large eye-diameter-to-ML ratio compared to some other squids, but that juveniles, which inhabit brightly lit waters, have a similarly high ratio to that observed in adults. This could be, in part, due to the fact that camera-type eyes must be a certain absolute size in order to be useful/functional [16]; therefore, juveniles may require proportionally “large” eyes (relative to other invertebrates with non-camera-type eyes) simply in order for these structures to function, while adults require even larger eyes (in absolute terms) to maximise light sensitivity, both down-welling from the surface and bioluminescent signals from other deep-sea animals, within their very dark environment [17].

Muntz indicated that small animals, which move relatively slowly, may require shorter focal distances because objects of interest are often closer to them (Kirschfeld, 1974; 1976, as cited in [16]). Other authors have noted that many predators probably rely on having greater visual ranges than their prey, enabling them to identify prey before being detected themselves (*e*.*g*., Nilsson, as cited in [1]). Perhaps a squid species belonging to a genus whose main repertoire of defense behaviours relies altering the animal’s appearance (for example, ballooning the mantle and filling it with ink [18]), as an alternative to taking rapid evasive action, thus requires better visual acuity than similarly sized squid more reliant on actively fleeing—the visual deception needs to occur early enough to confuse or deter a potentially attacking predator.

### Retinal structure

Although the cellular composition of the photoreceptor appears consistent throughout ontogeny, a change was observed in the proportions in the inner and outer photoreceptor lengths. In early ontogenetic stages, the lengths of the outer and inner photoreceptors were nearly equal, whereas the length of the outer photoreceptor increased dramatically in adults, comprising 89–97% of the total photoreceptor length (Table 1). Hypothetically, the onset of ontogenetic descent into deeper, darker habitats (a common life history trait in oceanic squids [19] could be partly triggered by the development of the eyes, and the lengthening of the photoreceptors—the increased sensitivity to light could drive maturing squid into lower strata, where less photic stimulation would occur. Alternatively, as small squid begin ranging into greater depths, changes in the retinal structure could be triggered by exposure to ever lower environmental light levels.

**Fig 4:**
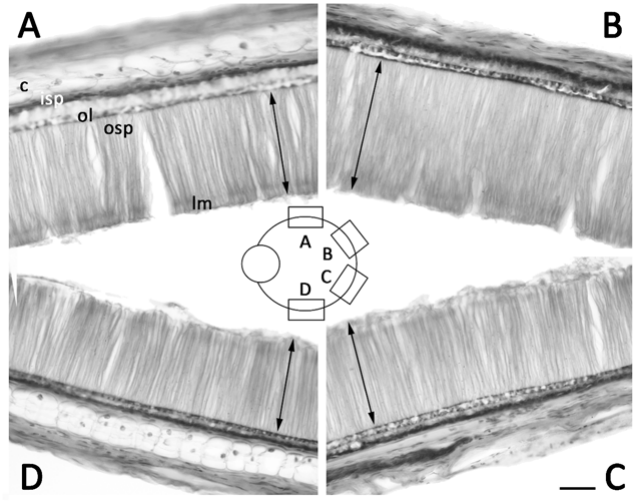
Cross-section at the equator of adult (ML 130 mm) *T*. *pellucida* retina showing no migrated ommin in any region and peripheral-central changes in photoreceptor thickness. Scale bar is 100μm. Arrows indicate length of outer photoreceptor segment. Abbreviations as in Figs 2 and 3.

Central photoreceptors were also longer than photoreceptors located toward the retina’s periphery in the majority of examined specimens (Fig 4). This was consistent with the findings of Matsui et al. [7], who observed that photoreceptor length is greatest in areas where the most light reaches the retina, and similar to observations on the vertebrate inverted retina, where photoreceptor length has been reported to change by region (central—peripheral) and as a function of light—dark adaptation [20]. In *T*. *pellucida*, the longer outer segments found centrally suggest that the retinal area directly in the axial length is structured to gather the most incoming light.

### Ommin migration

The screening layer of ommin located between the inner and outer photoreceptors does not appear to change in absolute thickness with growth of the specimen; in most samples, the thickness of this layer remained approximately 10μm. While ommin density within this layer was not specifically examined, no obvious ontogenetic changes were observed, suggesting that the ommin protecting the shorter photoreceptors of younger individuals (which are exposed to bright lighting conditions for an extended portion of each day) may, without drastic increase, also sufficiently protect both the longer photoreceptors of older animals (which are less frequently exposed to bright light). However, the primary long-term mechanism facilitating vision in the dark environments inhabited by adults seems to be increased photoreceptor length. It remains unknown whether photoreceptor length in squid could also change (on a smaller scale) over shorter time-frames—e.g., in response to diel vertical migration—as has been observed in some vertebrates whose eyes can make short-term light/dark adaptive adjustments [21].

Daw and Pearlman [5] first reported ommin mobility in the squid *Doryteuthis pealeii*: when introduced to light-intense conditions, some ommin migrated from the “normal” position (within the screening layer) to the outer edge of the photoreceptor, over the course of 5–15 minutes. Since the action spectrum of the ommin migration matches that of rhodopsin (the visual pigment) [5], this adaptation presumably reduces or prevents over-stimulation by shielding the inner receptors from excess incoming light [22]. After the light source was removed, the ommin began to return to the screening layer within several minutes [5].

Similar migrations appear to happen in the eyes of *Teuthowenia*—all smaller specimens had a darkly pigmented “layer” at the tip of the outer receptor (Fig 3B), which became sparser or was not observed in more mature specimens. Young *Teuthowenia* are most commonly collected between 0 and 200 meters, while older animals are usually encountered from deeper, aphotic waters ([11]; Fig 1C); the retina thus receives far more light during early ontogenetic stages. The changes in photoreceptor length observed as the animal matures partially account for the eye’s ability to retain advanced visual capabilities across these disparate light conditions. The migratory screening pigment granules likely provide an additional means of protecting the retina during the bright light conditions experienced by young individuals (which presumably also need the short-term flexibility to see well at night). The ommin’s migratory capability appears to be retained into adulthood, but is probably less frequently utilised in the deep sea, and the thresholds and time frames of the response to light stimulus may differ from those of younger individuals.

## Conclusion

*Teuthowenia pellucida* is an abundant species (based on collection data) that inhabits different photic zones during different life stages. Paralarvae and small juveniles reside in the epipelagic zone, with a habitat shift down into aphotic waters (below ~200 m) at around ML 50 mm. The eye diameter maintains a linear relationship with mantle length (a proxy of overall size) throughout ontogeny, which may be unusual among squids. The specific morphological changes observed in the retina of *T*. *pellucida* herein may coincide with the ontogenetic descent into deep-sea habitats; in particular, the outer portion of the photoreceptors increases dramatically in length as the animal matures, enabling the retina to collect more light in a darker environment. Migration of the screening pigment ommin appears to take place in this species, as has been reported in several other cephalopod taxa, but relatively little comparative information is available. To confirm these results, the eye growth and retinal structure of a wholly epipelagic or bathypelagic species (ideally both) should be examined throughout ontogeny for comparison. If these differ greatly from our observations on *T*. *pellucida*, the eye development reported here may characterise cephalopod species that exhibit ontogenetic descent from photic to aphotic marine habitats.

## References

[1] Warrant EJ, Locket NA. Vision in the deep sea. Biol Rev. 2004;79(3): 671–712. 15366767 10.1017/s1464793103006420

[2] Arnold JM. Closure of the squid cornea: a muscular basis for embryonic tissue movement. J Exp Zool. 1984;232: 187–195. 6502097 10.1002/jez.1402320206

[3] Boyle P, Rodhouse P. Cephalopods: ecology and fisheries. Oxford: Blackwell Publishing, 2005.

[4] Arnold JM. Normal embryonic stages of the squid, *Loligo pealii* (Lesueur). Biol Bull. 1965;128(1): 24–32.

[5] Daw NW, Pearlman AL. Pigment migration and adaptation in the eye of the squid, *Loligo pealei* . J Gen Physiol. 1974;63: 22–36. 4810208 10.1085/jgp.63.1.22 PMC2203541

[6] Gleadall IG, Ohtsu K, Gleadall E, Tsukahara Y. Screening-pigment migration in the octopus retina includes control by dopaminergic efferents. J Exp Biol. 1993;185(1): 1–16.

[7] Matsui S, Seidou M, Horiuchi S, Uchiyama I, Kito Y. Adaptation of a deep-sea cephalopod to the photic environment. J Gen Physiol. 1988;92: 55–66. 3171534 10.1085/jgp.92.1.55 PMC2228886

[8] Stavenga DG. Pigments in compound eyes In: Facets of vision. New York: Springer Berlin Heidelberg; 1989 pp. 152–172.

[9] Young JZ. Light- and dark- adaptation in the eyes of some cephalopods Proc zool Soc, Lond. 1963;140: 255–272.

[10] Evans AB. Ecology and ontogeny of the cranchiid squid *Teuthowenia pellucida* in New Zealand waters. MAppSc Thesis, Auckland University of Technology. 2013. Available: http://aut.researchgateway.ac.nz/handle/10292/5747.

[11] Voss NA. Systematics, biology, and biogeography of the cranchiid cephalopod genus *Teuthowenia* (Oegopsida). Bull Mar Sci. 1985;36(1): 1–85.

[12] Johnson B. *Vampyroteuthis infernalis*, 2000. Available: http://animaldiversity.ummz.umich.edu/accounts/Vampyroteuthis_infernalis.

[13] Nilsson DE, Warrant EJ, Johnsen S, Hanlon R, Shashar N. A unique advantage for giant eyes in giant squid. Curr Biol. 2012;22: 1–6 10.1016/j.cub.2011.12.009 22425154

[14] Sweeney AM, Haddock SHD, Johnsen S. Comparative visual acuity of coleoid cephalopods. Integr Comp Biol. 2007;47: 808–814. 10.1093/icb/icm092 21669760

[15] Imber MJ. The squid families Cranchiidae and Gonatidae (Cephalopoda: Teuthoidea) in the New Zealand region. New Zeal J Zool. 1978;5(3): 445–484.

[16] Muntz WRA. Visual systems, behaviour, and environment in cephalopods In: Archer SN, Djamgoz MBA, Loew ER, Partridge JC, Vallerga S, editors. Adaptive mechanisms in the ecology of vision. Springer Netherlands; 1999 pp. 467–483.

[17] Collin SP, Partridge JC. Retinal specializations in the eyes of deep-sea teleosts. J Fish Biol. 2006;49: 157–174.

[18] Dilly PN. *Taonius megalops*, a squid that rolls up into a ball. Nature. 1972;237: 403–404.

[19] Seibel BA, Goffredi SK, Thuesen EV, Childress JJ, Robison BH. Ammonium content and buoyancy in midwater cephalopods. J Exp Mar Biol Ecol. 2004;313(2): 375–387.

[20] Braekevelt CR. Photoreceptor fine structure in light- and dark-adaptation in the butterfly fish (*Pantodon buchholzi*). Anat Anz. 1990;171(5): 351–8. 2088152

[21] LaVail MM. Circadian nature of rod outer segment disc shedding in the rat. Invest Ophth Vis Sci. 1980;19(4): 407–411. 7358492

[22] Mäthger LM, Shashar N, Hanlon RT. Do cephalopods communicate using polarized light reflections from their skin? J Exp Biol. 2009;212: 2133–2140. 10.1242/jeb.020800 19561202

